# AI-CAD enhances pulmonary TB detection and yield in active case finding

**DOI:** 10.5588/ijtldopen.25.0088

**Published:** 2025-10-10

**Authors:** A. Frederick, R. Kubendiran, T. Neelagandan, K.K. Shankar, A. Ojha, R. Pant, S. Pardeshi, T. Gupte, A. Kharat

**Affiliations:** ^1^ Department of Health and Family Welfare, Chennai, India;; ^2^ DeepTek Medical Imaging Pvt. Ltd., Pune, India.

**Keywords:** tuberculosis, Tamil Nadu, India, artificial intelligence, computer-aided detection, chest X-ray

## Abstract

**BACKGROUND:**

India accounts for 27% of global TB incidence and bears the highest TB burden worldwide. This study evaluates the performance of an AI-assisted computer-aided detection (AI-CAD) solution in a community-based, active case-finding TB screening programme conducted in Tamil Nadu, India. It also provides a comparative analysis of AI-assisted screening and conventional screening methods.

**METHODS:**

Community-based TB screening was carried out using mobile diagnostic units equipped with digital X-ray machines. The performance of the AI-CAD solution was evaluated by calculating area under the receiver operating characteristic (AUROC) curve, sensitivity, and specificity. Additionally, data from five districts that used conventional screening methods were analysed for comparative analysis against AI-assisted screening.

**RESULTS:**

AI-CAD exceeded the World Health Organization (WHO)-recommended minimum target product profile (TPP) with a sensitivity of 0.93 (95% confidence interval [CI]: 0.88, 0.97) and a specificity of 0.83 (95% CI: 0.82, 0.83). AI interpretation was significantly associated with positive TB diagnosis (odds ratio: 58.95, *P* < 0.0001). AI-assisted screening led to a 2.09-fold increase in TB diagnoses (*P* < 0.05) and a 2.86-fold higher sputum positivity rate (*P* < 0.05) compared with the conventional screening approach.

**CONCLUSION:**

The AI-CAD met and exceeded the WHO’s minimal TPP for TB detection. The higher sputum-positive yield reinforces AI-CAD’s potential in large-scale TB screening programmes.

According to the 2024 World Health Organization (WHO) report, 8.16 million people were newly diagnosed with tuberculosis (TB) in 2023, an increase from 7.4 million in 2022.^1^ In India, TB remains a significant public health challenge. The India TB Report 2023 states that the country accounted for 27% of the global TB burden, with an estimated 2.82 million cases and a case-fatality ratio of 12% in 2022.^1^ These statistics highlight the urgent need for innovative approaches for TB detection and management in India. Beyond treating individuals affected by TB, it is necessary to prioritise interrupting transmission. TB can remain asymptomatic for extended periods, necessitating more than just passive case finding to control its spread.^2,3^ Active case finding (ACF) involves proactively reaching out to the community to identify undiagnosed infections and connect those individuals to appropriate care. Current evidence shows that ACF interventions are effective in detecting TB early, particularly in low-resource settings.^4–6^ This proactive approach not only helps early diagnosis but also plays a significant role in reducing TB transmission. Modern prevalence surveys indicate that up to 60% of people with TB do not report classical symptoms, prompting the use of chest X-rays (CXRs) as a triage or screening tool rather than solely a diagnostic tool.^7^ Recent developments in artificial intelligence (AI) have further enhanced CXR analysis, enabling unbiased visual interpretation without requiring highly trained human readers, who are often scarce in many settings.^8^ AI can analyse CXRs for cardiothoracic abnormalities, including those related to TB, potentially addressing current limitations and providing a significant advantage in settings where human resources are limited.^9–11^ AI systems reduce human inter-reader variability and provide radiologic services where radiologists are unavailable. The WHO’s consolidated guidelines on TB recommend that AI-assisted computer-aided detection (AI-CAD) systems should achieve at least 90% sensitivity and 70% specificity for detecting TB-related abnormalities on chest radiographs. Studies on the performance of AI-CAD for TB show that it matches or exceeds the accuracy of experienced medical doctors.^1^ In community-based ACF, AI-CAD systems could revolutionise TB screening by enabling large-scale, efficient, and accurate identification of individuals affected by TB. Integrating AI-CAD with digital radiography in mobile screening units could facilitate access to TB screening in remote and underserved areas, thereby increasing the reach and effectiveness of TB control programmes.^12,13^ Moreover, AI-CAD systems can provide immediate feedback, allowing quicker referral and treatment initiation, which is crucial in controlling TB spread.

In this study, we report findings from integrating commercially available AI-CAD software in ACF screening of TB in India. The primary objective of this study was to evaluate the performance of CAD software in detecting TB from chest radiographs of patients, using sputum results as the reference standard. A comparative analysis is also conducted to evaluate the differences in TB diagnosis between districts utilising CAD software and those using conventional screening methods. This research demonstrates the potential benefits of AI-CAD in community-based ACF initiatives, providing valuable insights into its applicability and effectiveness in real-world settings.

## METHODS

### Study design

**Figure 1.**
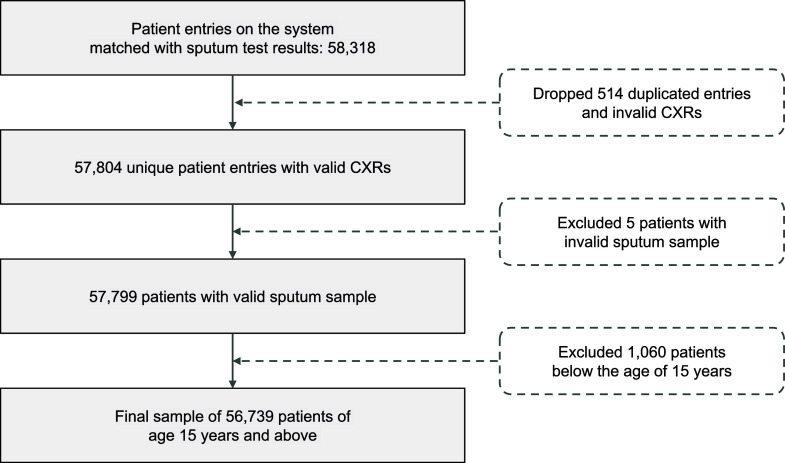
The flow chart depicts the inclusion and exclusion criteria for data curation and analysis.

This retrospective observational study evaluated the performance of Genki v3.4.2 (DeepTek.ai) for TB detection in a community-based screening programme across six districts of Tamil Nadu, India: Pudukkottai, Kanchipuram, Vellore, Salem, Tirunelveli, and Tiruchirappalli, using data from March 2023 to February 2024 (Figure 1). The screening in all districts was conducted as a part of the National TB Elimination Programme. Mobile diagnostic units (MDUs) with digital X-ray machines and Genki software were deployed in select districts. Before X-ray screening, information regarding the patient’s demographics, medical history, occupation, and TB symptoms was recorded. For comparative analysis, the study assessed the impact of AI on TB detection across 10 districts in Tamil Nadu. Five districts – Kanchipuram, Salem, Tiruchirappalli, Tirunelveli, and Vellore – were designated as AI intervention districts, while five other districts – Villupuram, Coimbatore, Madurai, Cuddalore, and Tirupur – served as control districts that followed the conventional method of screening (Supplementary Data Figure S1). Only individuals aged ≥15 years were included in the analysis. The inclusion and exclusion criteria of the study are depicted in Figure 1. The Institutional Human Ethics Committee (IEC), Directorate of Public Health and Preventive Medicine (Chennai, Tamil Nadu, India), approved this study. Due to the retrospective nature of the study, the requirement for written consent from participants was waived by the IEC.

### Reading and testing operations

DeepTek’s Genki software was deployed on-site within the MDUs. Each participant’s CXR was analysed by the software, generating a TB probability score ranging from 0 to 1. For this screening programme, the software was deployed at a predefined threshold of 0.22. Supplementary Data Figure S1 depicts the protocol followed at screening sites. Individuals who the software identified as potentially having TB or those experiencing symptoms suggestive of TB were referred for sputum testing using CBNAAT, TrueNat, or smear microscopy. Patients recommended by the medical officer were also subjected to sputum testing. Districts using the conventional method of screening relied on medical officers to identify clinical symptoms, interpret chest radiographs, and assess individual vulnerability to determine eligibility for sputum collection without the support of AI-based classification. Sputum test results were used as a reference standard to confirm TB diagnosis.

### Performance evaluation

AI’s performance for the overall study population was evaluated by calculating the AUROC, sensitivity, and specificity at a predefined threshold of 0.22. To evaluate Genki’s performance against the WHO’s target product profile (TPP) for community-based triage, we calculated the AI’s sensitivity at 70% specificity (the minimal TPP requirement) and 80% specificity (the optimal TPP requirement), respectively. 95% confidence intervals (CIs) for each of these performance measures were estimated using empirical bootstrapping. AI performance was also evaluated across demographic subgroups, including age, sex, past TB history, symptoms, patient source, comorbidities, lifestyle choices, and body mass index (BMI) categories. A forest plot was generated to visualise sensitivity and specificity for each demographic subgroup using the forest plot library in Python. Additionally, odds ratios were calculated to compare various factors against the sputum test results (Supplementary Data).

### Comparative analysis

For both groups, the percentage of X-rays acquired relative to the population was calculated. The percentage of X-rays that resulted in positive sputum tests was also determined. Additionally, the proportion of sputum samples collected from the total X-ray screenings, as well as the percentage of positive sputum tests, was assessed. Finally, the overall percentage of X-ray screenings that led to a confirmed TB diagnosis was calculated. The comparative analysis involved calculating fold changes to quantify differences in TB detection metrics between districts utilising AI-assisted screening and those using conventional methods. A Student’s *t* test was performed to determine the statistical significance of these differences. *P* < 0.05 was considered statistically significant.

## RESULTS

### Demographics and clinical characteristics of the screened population

We analysed the final dataset of 56,739 participants with their corresponding CXRs and medical data. See Supplementary Data for the demographic and clinical characteristics of participants screened in the programme. Across the six districts in Tamil Nadu, 12.36% of participants were from Kanchipuram, 17.43% from Pudukkottai, 18.52% from Salem, 14.28% from Tiruchirappalli, 20.39% from Tirunelveli, and 16.98% from Vellore. The mean and median ages of the population were 52.6 years (±14.7) and 54 years (interquartile range [IQR]: 43, 64), respectively. Of the total population, 32,778 (57.77%) were females and 23,945 (42.19%) were males. The majority of participants were aged between 45 and 75 years, where 22.89% were aged 45–55 years, 25.02% were aged 55–65 years, and 17.89% were aged 65–75 years. 1.71% of participants reported a history of TB. Common symptoms included cough, reported by 29.76% of participants, and chills, reported by 3.35%. 24,115 (42.50%) participants exhibited different comorbid conditions. 25.11% of participants had hypertension, 24.77% had diabetes, 4.61% suffered from bronchial asthma, and 4.43% had chronic obstructive pulmonary disease (COPD). Lifestyle choices such as alcohol consumption and tobacco use were reported by 12.51% and 16.87% of participants, respectively. The BMI of participants varied, with 11.02% of participants being underweight, 48.34% being healthy weight, 24.84% being overweight, and 9.75% being obese.

### Performance of CAD with sputum testing results as reference

**Figure 2.**
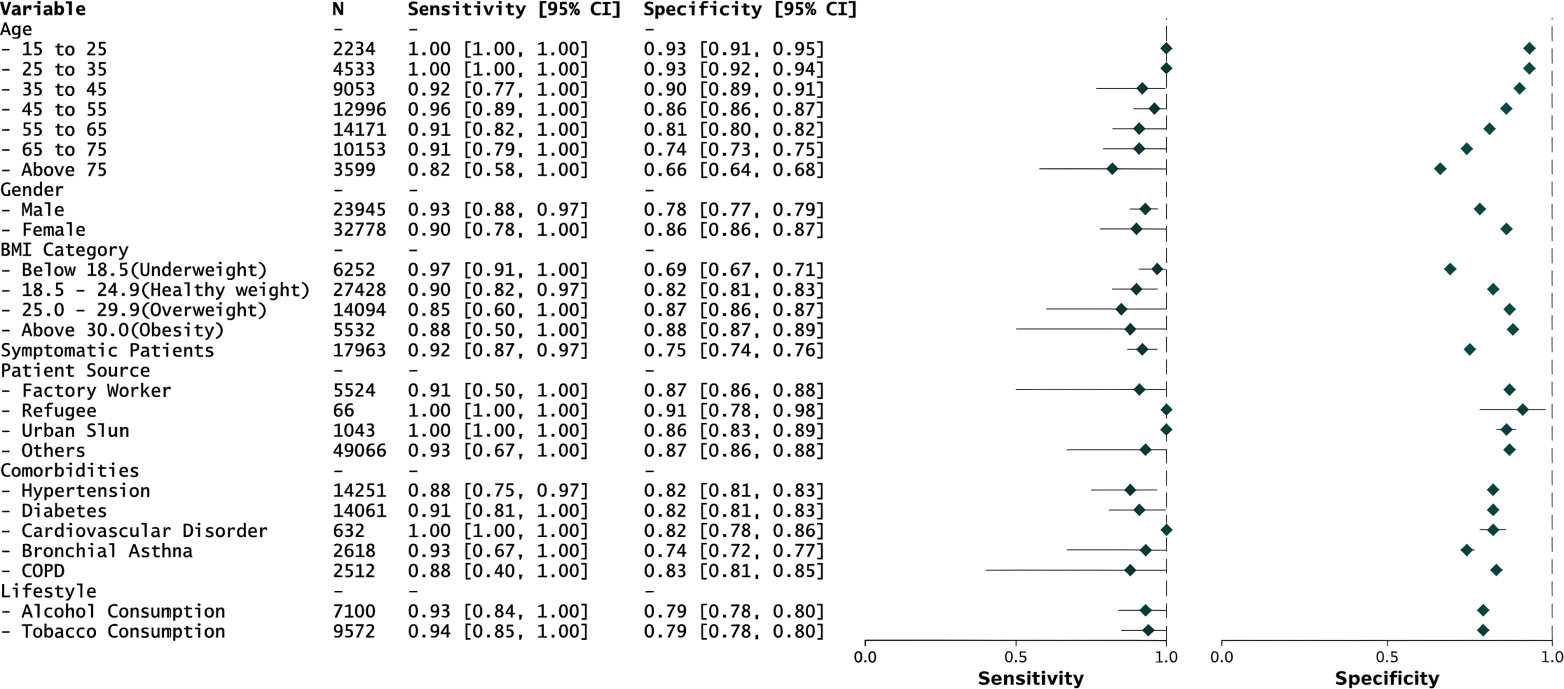
Model performance for different demographic categories. The forest plot on the right demonstrates the sensitivity and specificity values.

The overall performance of the model in all six districts surpasses the WHO-recommended minimum TPP with a sensitivity of 0.93 (95% CI: 0.88, 0.97), specificity of 0.83 (95% CI: 0.82, 0.83), and AUROC of 0.93 (95% CI: 0.92, 0.95) (Table 1). The performance across districts shows non-significant variability with AUROC ranging from 0.87 to 0.97 (Supplementary Data Figure S2). The model performs well across all age groups, with exceptional performance in the 15–25 years (sensitivity: 1.00, specificity: 0.93), 25–35 years (sensitivity: 1.00, specificity: 0.93), and 45–55 years (sensitivity: 0.96, specificity: 0.86) categories, where it exceeds the WHO-recommended optimal TPP. For the 35–45 years (sensitivity: 0.92, specificity: 0.90), 55–65 years (sensitivity: 0.91, specificity: 0.81), and 65–75 years (sensitivity: 0.91, specificity: 0.74) age groups, the model meets the WHO-recommended minimal TPP requirement. In the age group >75 years (sensitivity: 0.81, specificity: 0.66), the model’s performance decreases slightly (Figure 2).

**Table 1.** Performance of the AI model across six districts.

District	N	Sensitivity (95% CI)	Specificity (95% CI)	AUROC (95% CI)
Pudukkottai	9,893	0.960 (0.863, 1.000)	0.907 (0.899, 0.915)	0.963 (0.929, 0.983)
Kanchipuram	7,019	0.882 (0.75, 1.000)	0.789 (0.776, 0.802)	0.917 (0.869, 0.96)
Vellore	9,639	0.848 (0.667, 1.000)	0.858 (0.848, 0.868)	0.877 (0.798, 0.952)
Salem	10,510	0.905 (0.794, 1.000)	0.825 (0.814, 0.835)	0.928 (0.889, 0.959)
Tirunelveli	11,572	0.933 (0.778, 1.000)	0.783 (0.772, 0.793)	0.935 (0.865, 0.979)
Tiruchirappalli	8,106	1.000 (1.0, 1.0)	0.797 (0.784, 0.809)	0.970 (0.961, 0.979)
Overall	56,739	0.925 (0.877, 0.967)	0.828 (0.823, 0.832)	0.934 (0.915, 0.950)

CI = confidence interval; AUROC = area under the receiver operating characteristic.

The model shows comparable performance across genders, with a sensitivity of 0.93 and specificity of 0.78 for men and a sensitivity of 0.90 and specificity of 0.86 for women. However, there is a noted decrease in model performance in underweight, overweight, and obese patients compared with those of healthy weight (Supplementary Data). Even in patients with comorbidities, such as hypertension, cardiovascular disorders, diabetes, bronchial asthma, and COPD, the model performs at par with overall performance, demonstrating its generalisability (Figure 2). In summary, the AI model meets and, in some cohorts, exceeds the WHO’s minimal TPP requirements, showing promise for effective integration into TB detection workflows. Further refinements and validations, especially to enhance specificity without compromising sensitivity, could align the model closer to meeting optimal TPP standards.

### Performance against the WHO TPP reference

In this study, the software’s performance was assessed against the WHO-recommended TPP for TB detection (Supplementary Data). We evaluated the CAD software using several key metrics, including sensitivity, specificity, and the AUROC curve. The software demonstrated strong performance metrics, with an AUROC of 0.93 across all evaluated scenarios, indicating high discriminative ability. As shown in Supplementary Data, the software surpasses the WHO-recommended minimal TPP criteria (0.70 specificity) with 0.94 (95% CI: 0.90, 0.98) sensitivity. At the WHO-recommended optimal specificity (0.80), an overall sensitivity of 0.93 (95% CI: 0.88, 0.97) was achieved with CIs overlapping the optimal TPP. Negative predictive value (NPV) values remained exceptionally high (99.9%), ensuring that negative AI interpretations are highly reliable.

### Comparative analysis of AI-aided screening versus conventional screening

The comparative analysis of the two different screening methods revealed that X-ray screenings were 1.22 times more frequent in districts utilising AI compared with districts following the conventional method of screening. Notably, districts utilising AI-assisted screening demonstrated more efficient sputum collection, with a 0.91-fold frequency compared with districts following conventional screening, indicating a more targeted approach. This targeted strategy proved effective, as AI-assisted districts’ sputum positivity rate was significantly higher (2.86 fold, *P* < 0.05) (see Supplementary Data). While individuals diagnosed with TB by clinical assessment were slightly less frequent in districts utilising AI-assisted screening (0.87 fold), the overall TB diagnosis rate was 2.09 times higher (*P* < 0.05) (Supplementary Data Figure S4). The percentage of positive X-rays and sputum-positive samples was also higher in districts utilising AI-assisted screening, with fold changes of 2.35 and 3.15, respectively (*P* < 0.05). The total percentage positive was 1.72 times higher in districts utilising AI-assisted screening, further supporting the enhanced efficiency of the AI-assisted approach (Table 2).

**Table 2.** Difference in parameters recorded for conventional districts and AI districts.

Parameter	Total for conventional district (A)	Total for AI-assisted district (B)	Fold change (B/A)	*P*-value
Total population	16,345,626	17,843,712	1.09	0.148
Total X-ray taken	44,775	54,410	1.22	0.148
Number of sputum samples collected	18,000	16,337	0.91	0.338
% of sputum samples collected	40.20%	30.03%	0.75	0.202
Number of individuals with sputum-positive TB (a)	100	286	2.86	0.011
% of individuals with sputum-positive TB	0.56%	1.75%	3.15	0.006
% of individuals who had X-ray taken and had sputum-positive TB	0.22%	0.53%	2.35	0.03
Individuals with TB confirmed by clinician (b)	63	55	0.87	0.33
Total individuals diagnosed with TB (a + b)	163	341	2.09	0.018
% of individuals with final TB diagnosis	0.36%	0.63%	1.72	0.047

## DISCUSSION

**Figure 3.**
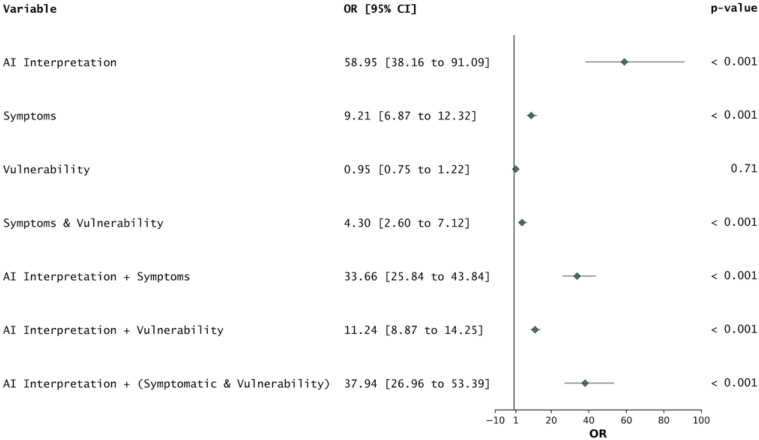
The association of AI predictions, symptoms, and vulnerability with sputum-positive TB depicted via an odds ratio calculation.

This study evaluated the effectiveness of integrating CAD software (DeepTek’s Genki) for TB detection within a community-based screening programme in Tamil Nadu, India. The CAD software’s overall performance, evaluated against the sputum test results, showed that it met and, in some cases, exceeded the WHO’s minimal TPP requirements for TB detection. The software’s high AUROC indicated its robust discriminative ability, reinforcing the potential of AI-assisted tools in large-scale TB screening programmes. Furthermore, AI interpretation shows a significant association with positive TB diagnosis, with an odds ratio of 58.95 (95% CI: 38.16–91.09, *P* < 0.0001). This indicates that the AI model is a strong predictor of TB when interpreting chest radiographs as compared with the association of symptoms with positive TB diagnosis, which showed an odds ratio of 9.21 (95% CI: 6.87–12.32, *P* < 0.0001) (Figure 3). In the comparative analysis of AI-assisted versus conventional screening, districts utilising AI-assisted screening demonstrated a significantly higher sputum positivity rate, 2.86 times greater than that observed in districts using conventional methods (Table 2). This indicates that the AI system was highly effective in identifying individuals with a higher likelihood of TB, leading to more targeted sputum collection. The more focused approach is further evidenced by the lower rate of sputum collection in these districts (0.91 fold), coupled with a higher percentage of positive sputum samples (3.15 fold). The overall TB diagnosis rate was 2.09 times higher in AI districts, demonstrating the significant impact of AI-assisted screening on case detection (Supplementary Data). This increased detection rate could have substantial public health implications, potentially leading to earlier treatment initiation and reduced community transmission.

Our study demonstrates the applicability and effectiveness of AI-based screening in real-world, resource-limited settings, enhancing its relevance for global TB control efforts. The integration of AI solutions like Genki in community-based ACF initiatives offers several advantages. AI can significantly enhance the efficiency and accuracy of TB screening, especially in remote and underserved areas where human resources are limited. One of the limitations of this study is that the data were collected from individuals who participated in the screening programmes. Information such as health, medical history, occupational status, and symptoms was self-reported and may introduce recall bias or inaccuracies. Any such inaccuracies could be carried further to the subgroup analyses. Future research should focus on long-term outcomes, including the impact on treatment initiation rates, treatment outcomes, and overall TB incidence in the community. In conclusion, this study provides strong evidence for the potential of AI-assisted ACF to significantly improve TB detection rates and screening efficiency in community-based settings. These findings support the integration of AI-CAD systems into TB screening programmes, particularly in high-burden countries like India, as a promising strategy to enhance TB control efforts.

## Supplementary Material


