# Supplementary material for: AI-CAD enhances pulmonary TB detection and yield in active case finding
Source: IJTLD Open. 2025 Oct 10;2(10):577–82. doi: 10.5588/ijtldopen.25.0088 (PMC12517255; doi:10.5588/ijtldopen.25.0088)

Title: AI-CAD Enhances Pulmonary Tuberculosis Detection and Yield in Active Case Finding: Evidence from Tamil Nadu, India

Authors: Asha Frederick[1] (Corresponding Author), Rambha Kubendiran[1], Tharageshwari Neelagandan[1], Kishore Kumar Shankar[1], Akanksha Ojha[2], Richa Pant[2], Siddhant Pardeshi[2], Tanveer Gupte[2], Amit Kharat[2]

Affiliations:
[1]Department of Health and Family Welfare, Chennai, Tamil Nadu, India
[2]DeepTek Medical Imaging, Pune, Maharashtra, India

Supplementary Methodology

District selection for AI-aided and Conventional screening:

As part of community screening programs aimed at diagnosing pulmonary TB in Tamil Nadu, six districts were selected for the implementation of AI-based screening. These districts were selected due to the dearth of qualified radiologists, making AI support particularly valuable. The districts with conventional screening were selected for comparative analysis based on comparable population sizes and prevalence. According to the 2021-2022 survey (Table S1), the average prevalence was comparable between the two groups, with 198 cases per 100,000 population in AI-screened districts and 236 cases per 100,000 population in conventionally screened districts. Since this screening was part of the National TB Elimination Programme (NTEP), all districts implemented similar awareness strategies and general initiatives to encourage people to volunteer for screening, ensuring that implementation initiatives did not bias the comparative analysis. Due to budget constraints, the AI-aided tool could not be extended to all districts.

Data Processing and Selection:

Data collected during the screening program encompassed AI-suggested results, sputum test results, and participants' demographics. The vulnerability information was collected and made available to MO/RO in both AI-aided and conventional screening settings. Patients were stratified based on geographical, clinical, and/or social vulnerability classification, following the active case-finding protocol. The inclusion and exclusion criteria of the study resulted in the removal of less than 1% of the data due to missing information. AI results included AI prediction based on the TB probability scores for each participant's CXR. In situations where the sputum test was negative or if sputum collection was not possible due to patient-related reasons, such as refusal, unwillingness to undergo an invasive procedure, or logistic challenges, the clinician made a decision regarding TB treatment based on clinical history and symptoms. This is particularly relevant in individuals with incipient TB or endobronchial TB. They were classified as individuals diagnosed with TB by clinical assessment. Demographic data included details such as age, sex, past TB history, reported symptoms, patient source (migrant, factory, miner, health care worker, prison, refugee, urban slum, other patient sources), presence of comorbidities, lifestyle choices, such as smoking or alcohol history, and BMI categories. This comprehensive

data collection allowed us to categorize participants and explore potential correlations or biases in AI's performance across different subgroups.

Odds Ratio Calculation:

In addition to assessing AI performance in detecting TB, the study also explored factors influencing the likelihood of a positive TB diagnosis (sputum test). Odds ratios were calculated to compare different factors against the sputum test results. These factors included AI Interpretation (AI suggests the presence of TB from a chest radiograph), symptoms suggestive of TB, vulnerability factors (comorbidities, high-risk patient source), and combinations of these factors. A forest plot was created to visualize these comparisons. All statistical analyses and graphical representations were conducted using Python version 3.6.10.

Supplementary Results

Distribution of individuals with sputum-positive TB across different demographic categories:
Based on symptoms, AI results, and on-ground medical officer/radiologist opinions, 24,200 participants were eligible for sputum testing. Sputum samples were collected from 16,593 of these participants, out of which 292 were confirmed TB positive based on the sputum testing (Supplementary Table 1). Of these 292 individuals, 270 were true positives (TP), where both AI and sputum test results were positive. Across the six districts in Tamil Nadu, Salem reported the highest number of individuals with TP-TB diagnosis i.e. 67 individuals (24.81%), followed by Tiruchirapalli with 54 individuals (20.00%), Pudukottai with 48 individuals (17.78%), Kanchipuram with 45 pateints (16.67%), Tirunelveli and Vellore with 28 individuals (10.37%) each. A significant gender disparity was observed. 223 (82.59%) were male, and only 47 (17.41%) were female. Age-wise, the highest true positives were noted in the 45-55 years and 55-65 years age groups, each having 27.04% of total individuals with a TP-TB diagnosis. This indicated an increased susceptibility to TB among the middle-aged and elderly population. The most common symptom among individuals with a TP-TB diagnosis was cough (77.78%), followed by weight loss (15.93%), chills (8.52%), fever (5.19%), night sweats (3.33%), and blood in sputum (0.74%). Comorbidities such as hypertension and diabetes were prevalent in 21.85% and 22.59% of the individuals with TP-TB, respectively. Lifestyle factors such as tobacco consumption (27.41%) and alcohol consumption (25.56%) were also significant among individuals with a TP-TB diagnosis. BMI distribution among these individuals showed that 44.07% had a healthy weight, 42.59% were underweight, 8.15% were overweight, and 2.59% were obese.

AI-assisted TB screening

The cumulative population of five districts utilizing AI-assisted screening was 17,843,712, out of which 54,410 X-ray screenings were conducted. These screenings resulted in the collection of 16,337 sputum samples, of which 286 tested positive for TB. Clinical evaluation identified 55 additional individuals with TB, bringing the total TB diagnoses to 341. X-ray screenings covered 0.30% of the population. Positive sputum results were found in 0.53% of the population screened using X-ray. Sputum was collected from 30.03% of the total X-ray screenings

conducted, with 1.75% testing positive. Overall, 0.63% of X-rays led to TB diagnoses (Supplementary Table 2).

Conventional TB screening

The districts following conventional screening had a cumulative population size of 16,345,626. X-ray screening was conducted for 44,775 participants. Sputum samples were collected from 18,000 participants, yielding 100 individuals with sputum-positive TB. The clinical evaluation identified 63 additional individuals with TB, totaling 163 TB diagnoses. X-ray screenings covered 0.27% of the population. Positive sputum results were found in 0.22% of X-rays. Sputum was collected from 40.20% of the total X-ray screenings, with 0.56% testing positive. Overall, 0.36% of X-rays led to TB diagnoses (Supplementary Table 3).

Odds ratio calculation

AI Interpretation shows a significant association with positive TB diagnosis, with an odds ratio of 58.95 (95% CI: 38.16 - 91.09, $p < 0.0001$). This indicates that the AI model is a strong predictor of TB when interpreting chest radiographs. For the presence of TB symptoms, the odds ratio was 9.21 (95% CI: 6.87 - 12.32, $p < 0.0001$), suggesting that symptomatic patients are significantly more likely to have a positive sputum test for TB. The vulnerability factor, which includes comorbidities and high-risk patient sources, did not show a significant association with positive TB diagnosis, with an odds ratio of 0.95 (95% CI: 0.75 - 1.22, $p = 0.709$). When combining symptoms and vulnerability, the odds ratio was 4.30 (95% CI: 2.60 - 7.12, $p < 0.0001$), indicating a significant association, albeit weaker than the presence of symptoms alone. The combination of AI Interpretation and symptoms yielded a very high odds ratio of 33.66 (95% CI: 25.84 - 43.84, $p < 0.0001$), showing that when both AI and symptoms suggest TB, the likelihood of a positive sputum test is greatly increased. The combination of AI Interpretation and vulnerability had an odds ratio of 11.24 (95% CI: 8.87 - 14.25, $p < 0.0001$), demonstrating a significant association. The combination of AI Interpretation with both symptoms and vulnerability resulted in an improved odds ratio of 37.94 (95% CI: 26.96 - 53.39, $p < 0.0001$), albeit showing a weaker association than that of AI interpretation and positive TB diagnosis. For individual demographic groups, the odds ratios were notably high across several age categories, with the 25-35 years age group showing the highest odds ratio of 374.60 (95% CI: 22.33 - 6283.44, $p < 0.0001$). Other significant results included the 45-55 years age group with an odds ratio of 153.19 (95% CI: 48.23 - 486.55, $p < 0.0001$) and the 35-45 years age group with an odds ratio of 96.81 (95% CI: 29.63 - 316.28, $p < 0.0001$). However, for age groups above 65 years, the odds ratios decreased, likely due to age-related changes in lung anatomy. Gender differences also revealed high odds ratios, with females having an odds ratio of 58.31 (95% CI: 23.18 - 146.69, $p < 0.0001$) and males with an odds ratio of 46.92 (95% CI: 28.63 - 76.91, $p < 0.0001$). Among patients with comorbidities, the odds ratios were significantly elevated for those with hypertension (OR: 33.22, 95% CI: 15.85 - 69.60, $p < 0.0001$), diabetes (OR: 46.88, 95% CI: 20.24 - 108.56, $p < 0.0001$), bronchial asthma (OR: 40.23, 95% CI: 5.28 - 306.51, $p < 0.001$), and COPD (OR: 33.86, 95% CI: 4.16 - 275.90, $p < 0.001$). Lifestyle factors also played a significant role, with alcohol consumption and tobacco use showing high odds ratios of 51.36 (95% CI: 20.68 - 127.54, $p < 0.0001$) and 55.59 (95% CI: 22.44 - 137.69, $p < 0.0001$), respectively.

Overall, the findings indicate that AI interpretation of chest radiographs greatly enhances the likelihood of detecting individuals with sputum-positive TB. The detailed odds ratios and their corresponding confidence intervals provide a comprehensive view of the predictive value of each factor and its combinations, as illustrated in the forest plot (Figure 3, Manuscript).

Tables

Table S1: Prevalence and prevalence-to-notification (P: N) ratio for selected districts.

Districts/State	TB prevalence (per 100,000 population) (95% CI)	TB P: N ratio (95% CI)
Tamil Nadu	212 (184 -239)	2.05 (1.8 -2.29)
**Districts with AI-aided screening**		
Kanchipuram	92 (12 -172)	1.27 (0 -2.58)
Salem	223 (129 -316)	2.35 (1.4 -3.22)
Tiruchirappalli	298 (176 -420)	3.03 (2.04 -3.94)
Tirunelveli	202 (132 -273)	2.08 (1.34 -2.76)
Vellore	176 (99 -253)	1.87 (1.13 -2.56)
**Districts with conventional screening**		
Viluppuram	189 (110 -267)	2.02 (1.47 -2.52)
Coimbatore	131 (48 -215)	1.82 (0.27 -3.25)
Madurai	486 (255 -718)	2.76 (1.54 -3.89)
Cuddalore	285 (92 -478)	2.6 (0.87 -4.18)
Tirupur	91 (0 -197)	0.95 (0 -1.95)

Table S2: The demographic characteristics of the population, including the number of people predicted to have TB by AI and diagnosed with TB by sputum test.

Group	Category	N (%)	AI Positive (%)	Sputum Positive (%)	AI Positive & Sputum Positive (TP) (%)
**Age Group**	15 to 25	2,234 (3.94%)	157 (1.57%)	12 (4.11%)	12 (4.44%)
	25 to 35	4,533 (7.99%)	273 (2.73%)	15 (5.14%)	15 (5.56%)
	35 to 45	9,053 (15.96%)	758 (7.58%)	36 (12.33%)	33 (12.22%)
	45 to 55	12,996 (22.90%)	1,447 (14.48%)	76 (26.03%)	73 (27.04%)
	55 to 65	14,171 (24.98%)	2,281 (22.82%)	80 (27.40%)	73 (27.04%)
	65 to 75	10,153 (17.89%)	2,219 (22.20%)	46 (15.75%)	42 (15.56%)

	75 above	3,599 (6.34%)	1,040 (10.40%)	27 (9.25%)	22 (8.15%)
**Sex**	Male	23,945 (42.20%)	4,505 (45.07%)	240 (82.19%)	223 (82.59%)
	Female	32,778 (57.77%)	3,668 (36.69%)	52 (17.81%)	47 (17.41%)
	Others	16 (0.03%)	2 (0.02%)	0 (0.00%)	0 (0.00%)
**TB History**	Past TB	972 (1.71%)	609 (6.09%)	39 (13.36%)	39 (14.44%)
	New presentation of TB	55,756 (98.27%)	7,565 (75.68%)	253 (86.64%)	231 (85.56%)
**Symptoms**	Cough	16,896 (29.78%)	3,853 (38.55%)	228 (78.08%)	210 (77.78%)
	Chills	1,900 (3.35%)	358 (3.58%)	24 (8.22%)	23 (8.52%)
	Fever	506 (0.89%)	124 (1.24%)	14 (4.79%)	14 (5.19%)
	Night Sweats	167 (0.29%)	55 (0.55%)	9 (3.08%)	9 (3.33%)
	Blood in Sputum	170 (0.30%)	59 (0.59%)	3 (1.03%)	2 (0.74%)
	Weight Loss	986 (1.74%)	338 (3.38%)	47 (16.10%)	43 (15.93%)
**Patient Source**	Migrant	223 (0.39%)	32 (0.32%)	0 (0.00%)	0 (0.00%)
	Factory Worker	5,524 (9.74%)	572 (5.72%)	11 (3.77%)	10 (3.70%)
	Miner	64 (0.11%)	12 (0.12%)	0 (0.00%)	0 (0.00%)
	Health Care Worker	949 (1.67%)	66 (0.66%)	0 (0.00%)	0 (0.00%)
	Prison	123 (0.22%)	18 (0.18%)	0 (0.00%)	0 (0.00%)
	Refugee	65 (0.11%)	7 (0.07%)	1 (0.34%)	1 (0.37%)
	Urban Slum	1,043 (1.84%)	102 (1.02%)	3 (1.03%)	3 (1.11%)
	Others	49,066 (86.48%)	7,395 (73.98%)	277 (94.86%)	256 (94.81%)
**Districts**	Kanchipuram	7,019 (12.37%)	1,293 (12.94%)	51 (17.47%)	45 (16.67%)
	Pudukottai	9,893 (17.44%)	716 (7.16%)	50 (17.12%)	48 (17.78%)
	Salem	10,510 (18.52%)	1,572 (15.73%)	74 (25.34%)	67 (24.81%)
	Tiruchirapalli	8,106 (14.29%)	1,394 (13.95%)	54 (18.49%)	54 (20.00%)
	Tirunelveli	11,572 (20.40%)	2,129 (21.30%)	30 (10.27%)	28 (10.37%)
	Vellore	9,639 (16.99%)	1,071 (10.71%)	33 (11.30%)	28 (10.37%)
**Comorbidities**	Hypertension	14,251 (25.12%)	2,119 (21.20%)	67 (22.95%)	59 (21.85%)
	Diabetes	14,061 (24.78%)	2,088 (20.89%)	67 (22.95%)	61 (22.59%)
	Cardiovascular Disorder	632 (1.11%)	94 (0.94%)	2 (0.68%)	2 (0.74%)
	Bronchial Asthma	2,618 (4.61%)	586 (5.86%)	15 (5.14%)	14 (5.19%)

	COPD	2,512 (4.43%)	342 (3.42%)	8 (2.74%)	7 (2.59%)
	Covid Recovered Patients	85 (0.15%)	8 (0.08%)	0 (0.00%)	0 (0.00%)
**Lifestyle**	Alcohol Consumption	7,100 (12.51%)	1,298 (12.99%)	74 (25.34%)	69 (25.56%)
	Tobacco Consumption	9,572 (16.87%)	1,726 (17.27%)	79 (27.05%)	74 (27.41%)
**BMI Category**	Below 18.5 (Underweight)	6,252 (11.02%)	2,017 (20.18%)	119 (40.75%)	115 (42.59%)
	18.5 – 24.9 (Healthy weight)	27,428 (48.34%)	5,052 (50.54%)	132 (45.21%)	119 (44.07%)
	25.0 – 29.9 (Overweight)	14,094 (24.84%)	1,910 (19.11%)	26 (8.90%)	22 (8.15%)
	Above 30.0 (Obesity)	5,532 (9.75%)	665 (6.65%)	8 (2.74%)	7 (2.59%)

Table S3: The performance parameters (sensitivity, specificity, and AUROC) are calculated at different specificity values following the WHO-recommended target product profile.

District	WHO recommendations	N	Sensitivity [95% CI]	Specificity [95% CI]	AUROC [95% CI]
Pudukottai	Minimal: Specificity 70%	9,893	0.980 [0.905, 1.000]	0.704 [0.69, 0.716]	0.963 [0.929, 0.983]
Kanchipuram		7,019	0.902 [0.765, 1.000]	0.704 [0.69, 0.719]	0.917 [0.869, 0.960]
Vellore		9,639	0.879 [0.706, 1.000]	0.704 [0.692, 0.717]	0.877 [0.798, 0.952]
Salem		10,510	0.932 [0.837, 1.000]	0.703 [0.691, 0.717]	0.928 [0.889, 0.959]
Tirunelveli		11,572	0.933 [0.778, 1.000]	0.704 [0.692, 0.715]	0.935 [0.865, 0.979]
Tiruchirapalli		8,106	1.000 [1.000, 1.000]	0.704 [0.689, 0.718]	0.970 [0.961, 0.979]
Pudukottai	Optimal: Specificity 80%	9,893	0.980 [0.905, 1.000]	0.804 [0.794, 0.816]	0.963 [0.929, 0.983]
Kanchipuram		7,019	0.882 [0.750, 1.000]	0.805 [0.792, 0.818]	0.917 [0.869, 0.960]
Vellore		9,639	0.848 [0.667, 1.000]	0.804 [0.793, 0.816]	0.877 [0.798, 0.952]
Salem		10,510	0.905 [0.794, 1.000]	0.805 [0.794, 0.815]	0.928 [0.889, 0.959]
Tirunelveli		11,572	0.933 [0.778, 1.000]	0.805 [0.794, 0.814]	0.935 [0.865, 0.979]
Tiruchirapalli		8,106	1.000 [1.000, 1.000]	0.805 [0.792, 0.818]	0.970 [0.961, 0.979]

Table S4: Districts Utilizing AI-Assisted Screening. This table presents various parameters recorded and calculated for districts that utilize the AI-assisted screening process.

AI Districts (N=5)						
**Parameter**	**Kanchipuram**	**Salem**	**Tiruchirappalli**	**Tirunelveli**	**Vellore**	**Total**
Total population	3,466,778	3,788,358	2,956,958	3,349,127	4,282,491	17,843,712
Total X-ray taken	8,252	11,360	11,873	12,126	10,799	54,410
Number of sputum samples collected	3,966	2,605	2,337	3,067	4,362	16,337
Number of individuals with sputum-positive TB	57	79	78	31	41	286
Individuals with TB confirmed by the clinician	4	27	11	11	2	55
Total number of individuals diagnosed with TB	61	106	89	42	43	341
% of the population that reported for X-ray screening	0.24%	0.30%	0.40%	0.36%	0.25%	0.30%
% of individuals who had an X-ray taken and had sputum-positive TB	0.69%	0.70%	0.66%	0.26%	0.38%	0.53%
% of sputum samples collected	48.06%	22.93%	19.68%	25.29%	40.39%	30.03%
% of individuals with sputum-positive TB	1.44%	3.03%	3.34%	1.01%	0.94%	1.75%
% individuals with final TB diagnosis	0.74%	0.93%	0.75%	0.35%	0.40%	0.63%

Table S5: Districts utilizing conventional screening methods: This table depicts various parameters recorded and calculated for districts using the conventional screening process.

Conventional Districts (N=5)						
**Parameter**	**Viluppuram**	**Coimbatore**	**Madurai**	**Cuddalore**	**Tirupur**	**Total**
Total population	3,756,984	3,756,984	3,302,067	2,831,464	2,698,127	16,345,626
Total X-ray taken	12,938	5,521	7,672	9,324	9,320	44,775
Number of sputum samples collected	3,838	4,275	4,585	1,346	3,956	18,000
Number of individuals with sputum-positive TB	10	14	38	12	26	100
Individuals with TB confirmed by the clinician	15	3	22	17	6	63
Total number of individuals diagnosed with TB	25	17	60	29	32	163
% of the population that reported for X-ray screening	0.34%	0.15%	0.23%	0.33%	0.35%	0.27%

% of individuals who had an X-ray taken and had sputum-positive TB	0.08%	0.25%	0.50%	0.13%	0.28%	0.22%
% of sputum samples collected	29.66%	77.43%	59.76%	14.44%	42.45%	40.20%
% of individuals with sputum-positive TB	0.26%	0.33%	0.83%	0.89%	0.66%	0.56%
% individuals with final TB diagnosis	0.19%	0.31%	0.78%	0.31%	0.34%	0.36%

Figures

Supplementary Figure 1:
The flowchart illustrates the workflow of AI-aided community screening and conventional screening for TB detection conducted in Tamil Nadu, India.

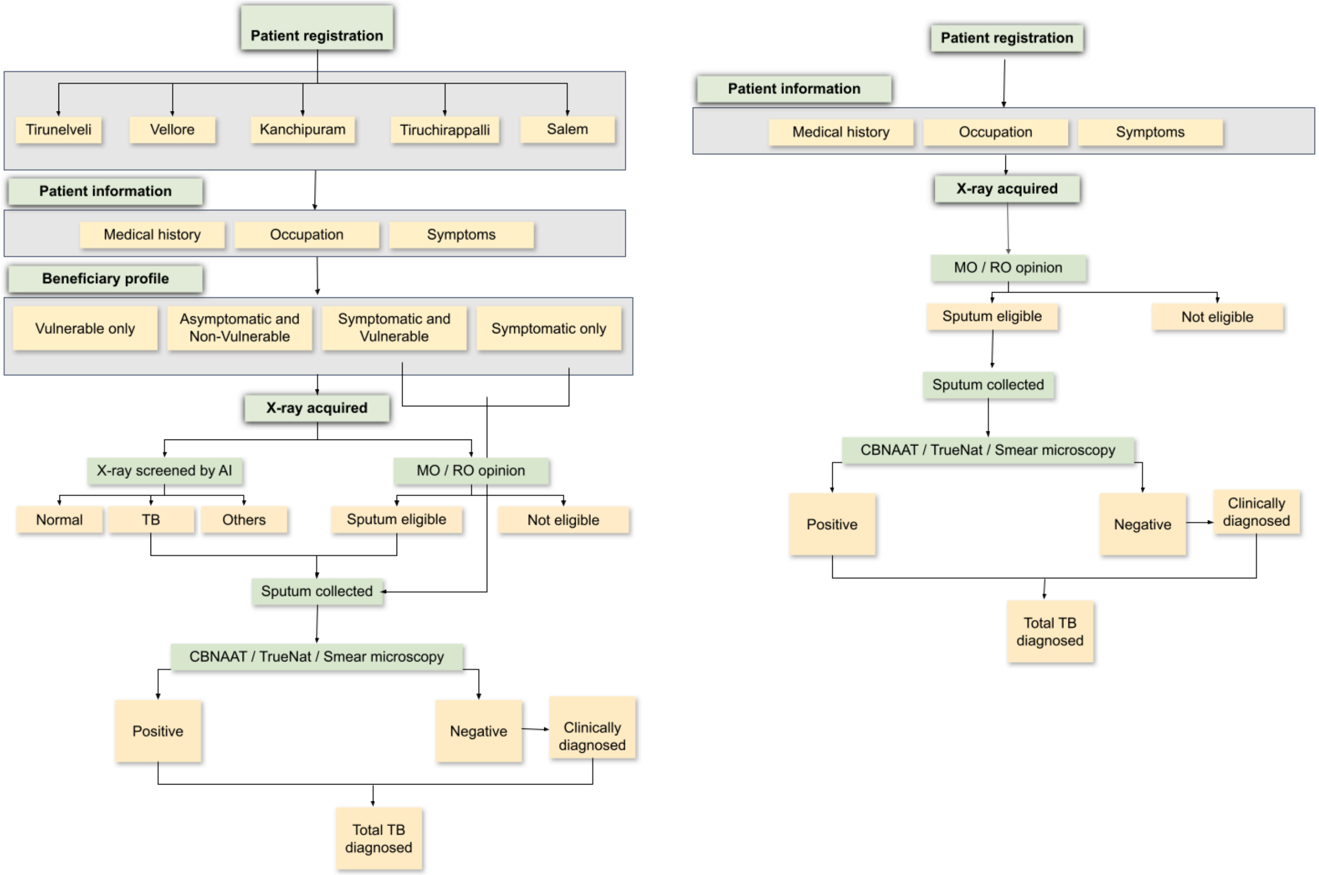

Supplementary Figure 2:
The area under the receiver operating characteristic (AUROC) for all the districts.

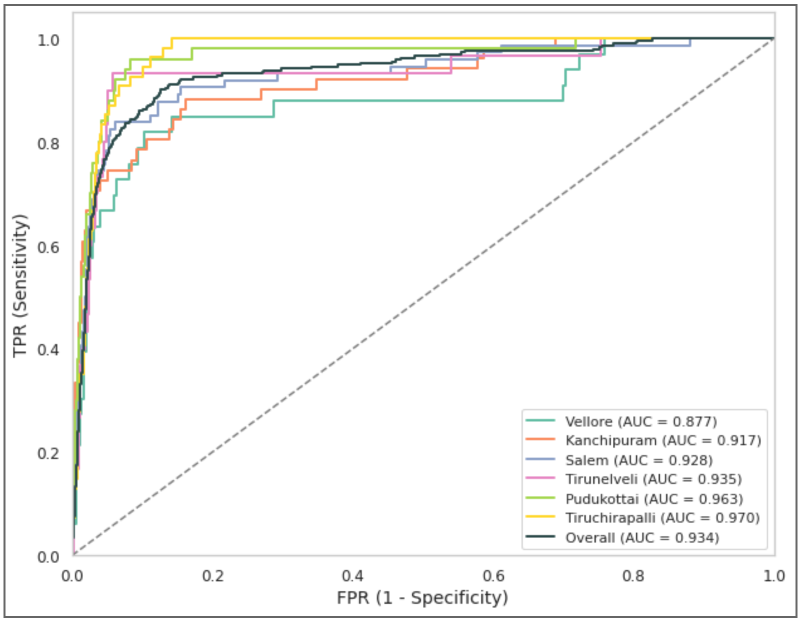

Supplementary Figure 3: The AUROC for various demographic categories to evaluate the model performance for each of the subgroups.

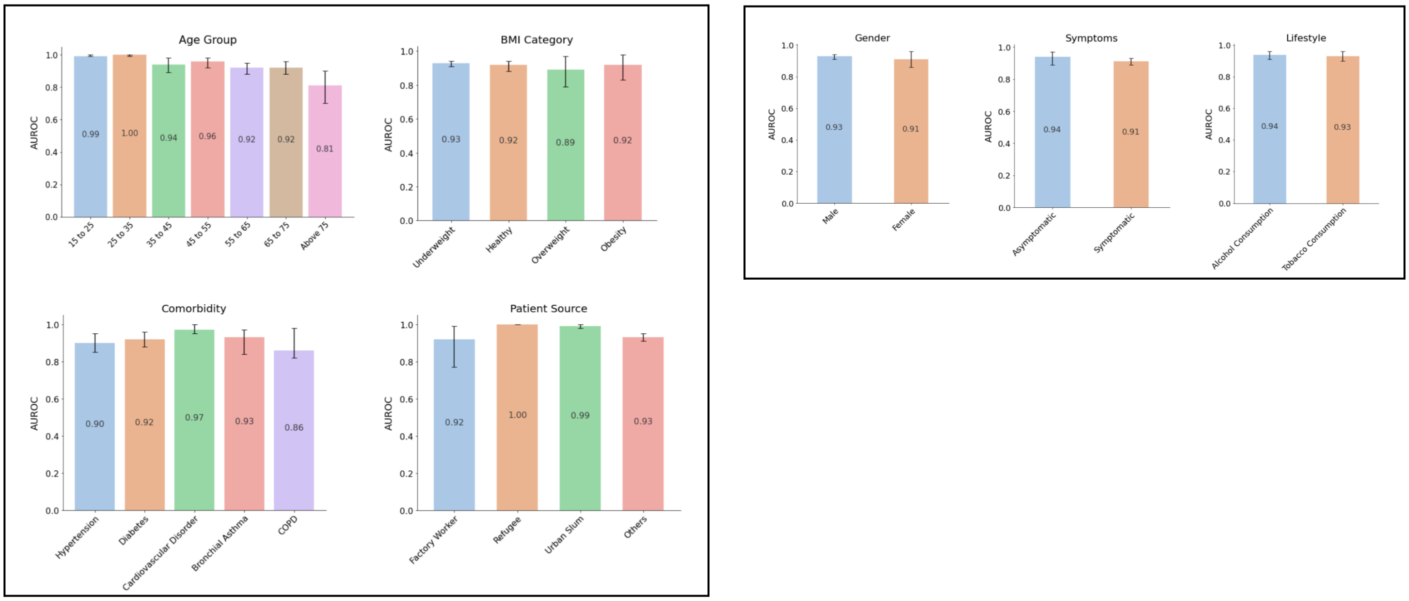

Supplementary Figure 4: Fold change observed in districts utilizing the AI-assisted screening method compared to districts using the conventional method. The figure illustrates the difference in various parameters between districts using AI-assisted screening and conventional screening methods. **p<0.05

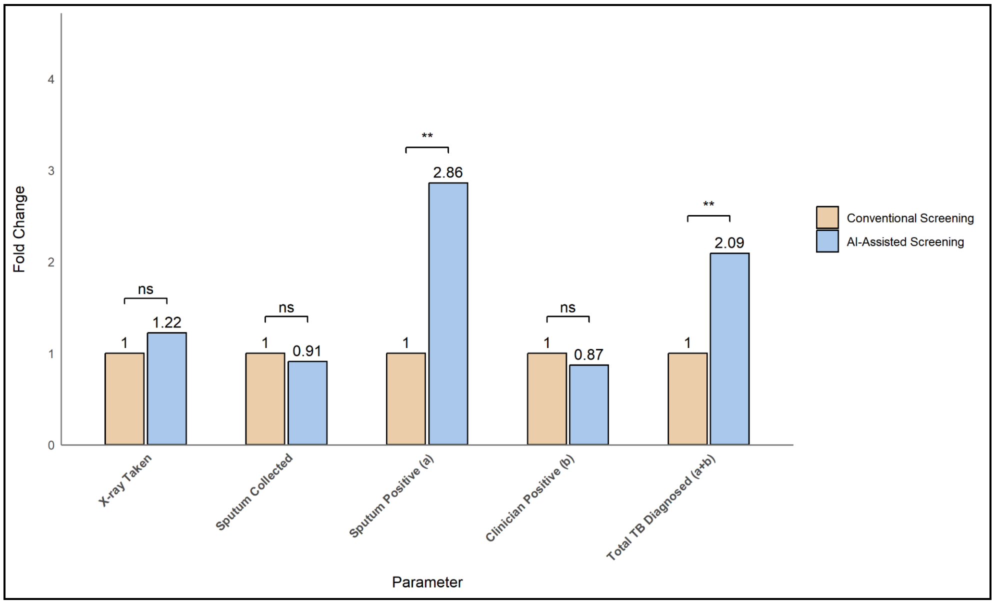

Supplement: Supplementary file 1 [file ijtldopen25-0088_supplementarydata1.pdf]
